# Effects of different photoperiods on melatonin level, cecal microbiota and breast muscle morphology of broiler chickens

**DOI:** 10.3389/fmicb.2025.1504264

**Published:** 2025-03-25

**Authors:** Miao Yu, Mengjie Xu, Guangju Wang, Jinghai Feng, Minhong Zhang

**Affiliations:** ^1^State Key Laboratory of Animal Nutrition and Feeding, Institute of Animal Sciences, Chinese Academy of Agricultural Sciences, Beijing, China; ^2^Adaptation Physiology Group, Wageningen University and Research, Wageningen, Netherlands

**Keywords:** photoperiod, melatonin, cecal microbiota, breast muscle morphology, inflammation

## Abstract

Long photoperiods are often characterized by enhanced oxidative stress-induced damage to skeletal muscle, reduced melatonin (MT) levels and intestinal microbiota dysfunction in broilers. In this study, we aimed to investigate the association of breast muscle morphology with melatonin levels and the cecal microbiota of broilers under different photoperiods. A total of 216 healthy 5-day-old Arbor Acres (AA) male broilers were randomly assigned to 12 L:12D, 18 L:6D and 24 L:0D photoperiods for 4 weeks (L = hours of light, D = hours of darkness). The concentration of inflammatory factors and MT concentrations was measured using ELISA kits, whereas breast muscle morphology was examined through the hematoxylin (H) and eosin (E) staining, and microbiota composition was identified through 16 s rRNA analysis. Extended light exposure significantly improved the growth rate of broilers, but significantly decreased feed efficiency (FE). Furthermore, it upregulated the concentration of IL-1β, IL-6 and TNF-*α* and induced an abnormal breast muscle morphology. Extended light exposure significantly decreased MT levels in the hypothalamus, cecum and breast muscle, while triggering the cecal microbiota composition disorder. Specifically, there was significant alteration to the dominant bacterial phylum, following exposure to long photoperiods, with the abundance of Firmicutes decreasing and the abundance of Bacteroidota increasing. Notably, the relative abundance of *Lactobacillus* showed a positive correlation with MT levels and a negative correlation with inflammatory cytokines. In conclusion, the present findings indicated that extended light exposure reduced the MT levels, which were related to disturbed cecal microbiota, damaging breast muscle morphology and inducing breast muscle inflammation in broilers.

## 1 Introduction

Long photoperiods, which are commonly applied in the modern and intensive broiler industry, aim to maximize the production efficiency through enhanced feed intake (Lewis and Gous, 2007). The photoperiods have also been reported to be critical factors in creating a suitable breeding environment to meet the increasing demand in the broiler chicken market. However, long photoperiods have been shown to induce detrimental effects on the quality of chicken products have been reported. For instance, it was reported that long photoperiods increased oxidative stress levels in the breast muscle of broilers (Li et al., 2010; Tuell et al., 2020), which may potentially damage the skeletal muscle morphology and induce atrophy (Zhang et al., 2023). Long photoperiods effectively increased the white striping occurrence of broilers, a muscle myopathy associated with oxidative stress (Gratta et al., 2023). Furthermore, photobiomodulation was found to enhance breast muscle atrophy in mice (Scalon et al., 2022). However, the influence of photoperiod treatments on breast muscle morphology is not completely understood.

Nighttime light exposure was demonstrated to decrease melatonin (MT) synthesis and secretion in broilers (Yang et al., 2022; Zhao et al., 2019; Saito et al., 2005). Notably, MT is a photoperiod-regulated hormone, involved in the transmission of photoperiodic signals to the peripheral organs (Tiwari et al., 2023). Understanding how the MT pathway regulates peripheral organ function in response to diverse photoperiods is a critical frontier in biological research. Some studies postulated that MT may alter skeletal muscle growth in birds and mammals: improving the myofiber formation of chick embryos (Bai et al., 2019), enhancing lipid mobilizing action in the skeletal muscle of the pigeon (John and George, 1976) and increasing the RNA content in the breast muscle of Japanese quail *Coturnix coturnix japonica* (Zeman et al., 1993). In mice, MT plays a role in the repair of skeletal muscle morphology injury (Su et al., 2023; Salagre et al., 2023), and, in rats, it accelerates the repair of muscle injury to promote skeletal muscle development and growth (Duan et al., 2022; Salucci et al., 2021). MT maintains the homeostasis of gut microbiota has been shown by influencing the intestinal bacterial community in broilers (Wang et al., 2020). Specifically, MT altered microbiota diversity, composition and function in geese (Li et al., 2020) and improved ileal morphology, barrier function, short-chain fatty acid (SCFA) profile, and microbial flora in laying ducks (Cui et al., 2022). Exogenous melatonin supplementation attenuated the intestinal microbiota disruption and restored normal intestinal microbiota function in mice (Li et al., 2023; Li et al., 2023).

Studies have uncovered that gut microbiota imbalance is often positively correlated with stress (Clavijo and Flórez, 2018). Exposure to different photoperiods induced alterations of the gut microbiota composition in broilers and mice (Oyola et al., 2021; Wang et al., 2018). Elsewhere, it was found that endogenous gut microbes studies have found that endogenous gut microbes regulated skeletal muscle growth in broilers (Jiang et al., 2023). In broiler, intestinal microbiota plays an important role in skeletal muscle development by modulating body metabolism and immunity (Zhang et al., 2022; Yin et al., 2024; Zhang et al., 2022). There is evidence that gut microbiota may regulate wooden breast myopathy by fine-tuning the dynamic changes in digestive metabolites in broilers (Kang et al., 2022). Moreover, breast muscle metabolites were strongly associated with various aspects of cecal microbes in broilers (Feng et al., 2022; Wen et al., 2023). However, research on the association of melatonin and cecal microbiota with the morphology of breast muscle under different photoperiods is limited. In this study, we speculated that melatonin and gut microbiota might be potential regulatory pathways mediating the effects of photoperiods. The aim of this study was to investigate the association between MT and cecal microbiota under extended light exposure and explore the potential regulatory pathways by which different photoperiods affect breast muscle morphology of broilers.

## 2 Materials and methods

The study was approved by the Institutional Ethics Committee of Experiment Animal Welfare and Ethics at the Institute of Animal Science of Chinese Academy of Agricultural Sciences (CAAS) (permit number: IAS 2022–117), and all the methods were carried out in accordance with relevant Institutional guidelines and regulations. And this study was confirmed that all methods were carried out in accordance with the guidelines and regulations of The ARRIVE guidelines 2.0.

### 2.1 Birds and experimental design

A total of 216 Arbor Acres (AA) male broilers (5-day old) with similar body weight (75 g ± 10) were randomly assigned to 3 photoperiod treatments (SD, LD and FD) with 6 replicates per treatment, with 12 broilers per replicate. The 3 groups (SD, LD and FD) received different photoperiods with 12 L:12D (SD, 12 h light), 18 L:6D (LD, 18 h light) and 24 L:0D (FD, 24 h light) for 4 weeks, respectively. The bird houses illuminated with LED lighting with 20 lux light intensity. All groups (SD, LD, and FD) received a standard corn and soybean meal basal diet in three feeding programs (5–7 days-old, 8–20 days-old and 21–32 days-old) (Table 2), formulated according to AA broiler recommendations (SCRIBD, 2019). The formal experimental period lasted 4 weeks (from 5 to 32 day of age). Broilers were housed in stainless steel cages without roofs (0.82 m width×0.70 m length×0.60 m height). The temperature and humidity shall be carried out according to the standard of the AA Broiler Feeding Management Manual. All broilers were farmed in different artificial climate chambers with the same size (4.08 m × 2.88 m × 2.38 m) of State Key Laboratory of Animal Nutrition and Feeding, Chinese Academy of Agricultural Sciences. Except for the photoperiods in the artificial climate chambers, other environmental parameters remained the same. Broilers were allowed free access to experimental diets and water (Table 1).

**Table 1:** Composition and nutrient levels of basic diets.

Items	5–7 days-old	8–20 days-old	21–32 days-old
Ingredient	Content (%)		
Corn	51.44	54.08	56.85
Soybean meal	40.21	36.82	33.86
Soybean oil	3.94	5.00	5.50
Limestone	1.00	0.85	0.90
CaHPO_4_	1.89	1.80	1.50
NaCl	0.30	0.30	0.30
DL-Methionine	0.21	0. 19	0. 19
L-Lysine	0.36	0.32	0.30
L-Threonine	0. 15	0. 14	0. 10
Premix^1^	0.50	0.50	0.50
Total	100	100	100
Nutrient levels^2^			
ME/(MJ/Kg)	2,961	3,038	3,095
CP (%)	22. 55	21.18	19.97
CF (%)	10.54	10.38	11.35
Ca (%)	0.94	0.85	0.79
AP (%)	0.43	0.41	0.35
Lysine (%)	1.46	1.34	1.25
Methionine (%)	0.54	0.51	0.49
Methionine+ cysteine (%)	0.92	0.87	0.84

ME, metabolizable energy; CP, crude protein; CF, crude fat; AP, available phosphorus. ^1^Premix provided the following per kg of the diet: 5–7 days-old: vitamin A 12000 IU, vitamin D_3_ 5,000 IU, vitamin E 80 mg, vitamin K_3_ 3.2 mg, vitamin B_1_ 3.2 mg, vitamin B_2_ 8.6 mg, vitamin B_6_ 4.3 mg, vitamin B_12_ 17 μg, pantothenic acid calcium 20 mg, nicotinic acid 65 mg, folic acid 2.2 mg, biotin 0.22 mg, choline 1,020 mg, Cu (CuSO_4_·5H_2_O) 16 mg, Fe (FeSO_4_·7H_2_O) 20 mg, Zn (ZnSO_4_·7H_2_O) 110 mg, Mn (MnSO^4^·H_2_O) 120 mg, Se (Na_2_SeO_3_) 0.3 mg, I (KI) 1.25 mg; 8–20 days-old: vitamin A 10000 IU, vitamin D_3_ 4,500 IU, vitamin E 65 mg, vitamin K_3_ 3.0 mg, vitamin B_1_ 2.5 mg, vitamin B_2_ 6.5 mg, vitamin B_6_ 3.2 mg, vitamin B_12_ 17 μg, pantothenic acid calcium 18 mg, nicotinic acid 60 mg, folic acid 1.9 mg, biotin 0.18 mg, choline 1,020 mg, Cu (CuSO_4_·5H_2_O) 16 mg, Fe (FeSO_4_·7H_2_O) 20 mg, Zn (ZnSO_4_·7H_2_O) 110 mg, Mn (MnSO_4_·H_2_O) 120 mg, Se (Na_2_SeO_3_) 0.3 mg, I (KI) 1.25 mg; 21–32 days-old: vitamin A 9000 IU, vitamin D_3_ 4,000 IU, vitamin E 55 mg, vitamin K_3_ 2.2 mg, vitamin B_1_ 2.2 mg, vitamin B_2_ 5.4 mg, vitamin B_6_ 2.2 mg, vitamin B_12_ 11 μg, pantothenic acid calcium 15 mg, nicotinic acid 45 mg, folic acid 1.6 mg, biotin 0.15 mg, choline 950 mg, Cu (CuSO_4_·5H_2_O) 16 mg, Fe (FeSO_4_·7H_2_O) 20 mg, Zn (ZnSO_4_·7H_2_O) 110 mg, Mn (MnSO_4_·H_2_O) 120 mg, Se (Na_2_SeO_3_) 0.3 mg, I (KI) 1.25 mg. ^2^ME determination was performed in the State Key Laboratory of Animal Nutrition and Feeding according to the bionic digestive Operation manual SDS3, CP content determination was conducted by using a Kjeldahl nitrogen analyzer and CF content determination was conducted by using a Soxhlet extractor. Other Nutrient levels were calculated value according to the Tables of Feed Composition and Nutritive Values in China (2022).

**Table 2:** Effects of different photoperiods on the performance of broilers.

Item	SD	LD	FD	SEM	*p* value
1-14d					
Average body weight/g	679.56^b^	781.89^a^	794.21^a^	12.89	<0.001
ADG/g	42.74^b^	50.10^a^	50.90^a^	0.93	<0.001
ADFI/g	45.07^c^	53.21^b^	65.10^a^	2.02	<0.001
FE	0.95^a^	0.94^a^	0.78^b^	0.02	<0.001
Right breast muscle mass/g	50.35^b^	62.81^a^	66.43^a^	1.89	<0.001
Right breast muscle mass-body mass ratio/%	7.31^c^	7.89^b^	8.51^a^	0.15	0.001
15-28d					
ADG/g	91.17^b^	101.01^a^	101.53^a^	1.32	<0.001
ADFI/g	113.55^c^	137.27^b^	152.10^a^	3.94	<0.001
FE	0.80^a^	0.74^b^	0.67^c^	0.01	<0.001
Right breast muscle growth/g	153.85^b^	173.83^a^	182.60^a^	3.99	0.003
Right breast muscle growth-body growth ratio/%	12.13	12.43	12.72	0.93	0.576
1-28d					
Average body weight/g	1956.00^b^	2195.99^a^	2215.60^a^	29.71	<0.001
ADG/g	68.21^b^	76.76^a^	77.47^a^	1.06	<0.001
ADFI/g	78.15^c^	93.69^b^	101.50^a^	2.40	<0.001
FE	0.87^a^	0.82^b^	0.76^c^	1.18	<0.001
Right breast muscle mass/g	204.20^b^	236.65^a^	249.03^a^	5.32	<0.01
Right breast muscle mass-body mass ratio/%	10.44^b^	10.78^ab^	11.24^a^	0.16	0.109

ADG, average daily gain; ADFI, average daily feed intake; FE, feed efficiency. ^a,b^Within a row, values with different superscripts indicate the significant difference (*p* < 0.05).

### 2.2 Sample collection

On 14 d and 28 d, one broiler from each replicate was selected with body weights close to the average after 12 h of feed deprivation. Then, the broilers from each group were killed by carbon dioxide (CO_2_). Tissue samples of the hypothalamus and breast muscle rinsed with sterile normal saline (NaCl 9 g/L) were immediately collected and snap-frozen in liquid nitrogen, then kept in a − 80°C freezer for measurements of gene expression and biochemical analysis. Another part of the breast muscle was immobilized by 4% paraformaldehyde to hematoxylin–eosin (HE) staining for histomorphological analysis. Cecal contents were immediately collected at d 28, and snap-frozen in liquid nitrogen, then kept in a − 80°C freezer for high-throughput sequencing and further analysis.

### 2.3 Performance

On 14 d and 28 d, the provided and residual feed amount, and broiler body weight of each replicate were recorded. The average body weight, average daily gain (ADG), average daily feed intake (ADFI), and feed efficiency (FE, FE = ADG/ADFI) were calculated. The right breast muscle of 1 broiler chicken from each replicate was randomly selected to determine the right breast muscle weight and right breast muscle mass-body mass ratio.

### 2.4 Inflammatory cytokines analysis of the breast muscle

The interleukin (IL)-1β (ZB00001DU, Zhongshang Boao Biotechnology Co., Ltd., Beijing, China), IL-6 (ZB00006DU, Zhongshang Boao Biotechnology Co., Ltd., Beijing, China) and tumor necrosis factor (TNF)-*α* (KJEIA0018D, Kangjia Hongyuan Biotechnology Co., Ltd., Beijing, China) concentrations in breast muscle were measured using ELISA kits by the Multiskan MK3 microplate reader (Thermo Fisher Scientific, Massachusetts, United States) according to the manufacturer’s instructions. Transfer the breast muscle into a glass homogenizer and add 5–10 mL of pre-cooled PBS buffer (1: 5 mass/volume ratio of tissue to PBS buffer is recommended) for thorough grinding, centrifuge the prepared homogenate at 3500 r/min for 15 min, and then retain the supernatant for assay (Yu et al., 2024). All the kits are chicken-specific. The intra-assay coefficient and the inter-assay coefficient of variation were 5 and 10%, respectively.

### 2.5 Breast muscle histology analysis

4% Paraformaldehyde-fixed breast muscle were embedded in paraffin (KD-BMIV biological tissue embedding machine, KEDEE, Jinhua, China) after being trimmed, dehydrated, transparentized, and waxed. After the wax block was made, the samples were sectioned (KD-2268 paraffin microtome, KEDEE, Jinhua, China) and then stained with hematoxylin (H) and eosin (E). The sections of each tissue were observed using a panoramic scanner (PANORAMIC® 250 Flash III DX, 3DHISTECH Ltd., Budapest, Hungary) and photographed.

### 2.6 MT concentrations analysis of hypothalamus and cecum

The concentrations of MT in the hypothalamus and cecum were measured using ELISA kits (CEA908Ge, Kangjia Hongyuan Biotechnology Co., Ltd., Beijing, China) by the Multiskan MK3 microplate reader (Thermo Fisher Scientific, Massachusetts, United States) according to the manufacturer’s instructions. The sample handling procedure was the same as described in 2.4. This kit was specifically used to detect MT and showed no obvious cross-reactivity with other similar substances. This kit was suitable for the pan-species (general) in all tissues. The intra-assay coefficient and the inter-assay coefficient of variation were 5 and 10%, respectively.

### 2.7 DNA extraction and 16 s rRNA analysis

Total microbial genomic DNA was extracted from cecal content samples using the E.Z.N.A.® soil DNA Kit (Omega Bio-tek, Norcross, GA, U.S.) according to the manufacturer’s instructions. The quality and concentration of DNA were determined by 1.0% agarose gel electrophoresis and a NanoDrop2000 spectrophotometer (Thermo Scientific, United States) and kept at −80°C before further use. The hypervariable region V3-V4 of the bacterial 16S rRNA gene was amplified with primer pairs 338F (5’-ACTCCTACGGGAGGCAGCAG-3′) and 806R (5’-GGACTACHVGGGTWTCTAAT-3′) (Liu et al., 2016) byT100 Thermal Cycler PCR thermocycler (BIO-RAD, United States). The PCR product was extracted from 2% agarose gel and purified using the PCR Clean-Up Kit (YuHua, Shanghai, China) according to manufacturer’s instructions and quantified using Qubit 4.0 (Thermo Fisher Scientific, USA). Purified amplicons were pooled in equimolar amounts and paired-end sequenced on an Illumina PE300/PE250 platform (Illumina, San Diego, USA) according to the standard protocols by Majorbio Bio-Pharm Technology Co. Ltd. (Shanghai, China). Raw FASTQ files were de-multiplexed using an in-house perl script, and then quality-filtered by fastp version 0.19.6 and merged by FLASH version 1.2.7. To minimize the effects of sequencing depth on alpha and beta diversity measure, the number of 16S rRNA gene sequences from each sample were rarefied to 20,000, which still yielded an average Good’s coverage of 99.09%, respectively.

### 2.8 Statistical analysis

All the results from the experiment were analyzed by using the 1-way ANOVA, performed using SPSS 23.0 (SPSS Inc., Chicago, IL). GraphPad Prism 8.0 (GraphPad Inc., San Diego, CA) was used for drawing. Replicate (*n* = 6) served as the experimental unit. The results in the tables are shown with the mean ± standard error of the mean (SEM). The *p* > 0.05, and *p* < 0.05 were deemed the statistical non-significance and significance, respectively. Bioinformatic analysis of the cecal content microbiota was carried out using the Majorbio Cloud platform.^1^ The taxonomy of each OTU representative sequence was analyzed by RDP Classifier version 2.2 against the 16S rRNA gene database (eg. Silva v138) using confidence threshold of 0.7. Based on the OTUs information, rarefaction curves and alpha diversity indices were calculated with Mothur v1.30.1 (Schloss et al., 2009). The similarity among the microbial communities in different samples was determined by principal coordinate analysis (PCoA) based on Bray–curtis dissimilarity using Vegan v2.5–3 package. The PERMANOVA test was used to assess the percentage of variation explained by the treatment along with its statistical significance using Vegan v2.5–3 package. A correlation between two nodes was considered to be statistically robust if the spearman’s correlation coefficient over 0.6 or less than −0.6, and the *p* value less than 0.01.

## 3 Results

### 3.1 Effects of different photoperiods on growth performance

As shown in Table 2, long photoperiods had significant effects on the ADG and ADFI (*p* < 0.05). Furthermore, in the FD group, FE was significantly reduced (*p* < 0.05). In the 1–14 days of the experiment, the LD group and the FD group broilers had a significant increase in the ADG and ADFI compared to those in the SD group, with increases of 17.33 and 19.33%, and 18.07 and 22.37%, respectively. Additionally, compared with the SD group, the right breast muscle mass (*p* < 0.05) and right breast muscle mass-body mass ratio (*p* < 0.05) were significantly increased due to long photoperiods. However, there was a significant decrease in FE in the LD group and the FD group by 17.38 and 16.81% compared to the SD group, respectively (*p* < 0.05). During weeks 3 to 4 of the experiment, the LD group and the FD group had significantly higher ADG and ADFI than the SD group (*p* < 0.05), while the FE of the LD group and the FD group significantly decreased by 8.26 and 16.77% compared to that of the SD group, respectively. The increasing light time had a significant effect on right breast muscle growth, with a 14.90 and 20.75% increase in the LD group and the FD group, respectively (*p* < 0.05), while there was no significant effect on the right breast muscle growth-body growth ratio (*p* = 0.576). Additionally, compared with the SD group, both the LD group and the FD group significantly increased the ADG and ADFI over the 4 weeks (*p* < 0.05), but the LD group and the FD group had a significant decrease in the FE (*p* < 0.05). On day 28 of the trial, both the LD group and the FD group significantly increased the right breast muscle mass compared to the SD group (*p* < 0.05). However, only the right breast muscle mass-body mass ratio of the FD group significantly increased, whereas that of the LD group had no significant effect compared to the SD group (*p* = 0.109). These data suggest that the long photoperiods led to a substantial improvement in growth rate, with a significant decrease in the FE.

### 3.2 Effects of different photoperiods on MT levels of hypothalamus and cecum

**Figure 1:**
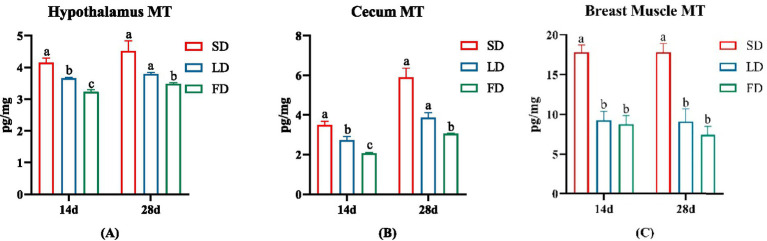
Effects of different photoperiods on MT concentrations of broilers. **(A)** Hypothalamus. **(B)** Cecum. **(C)** Breast muscle. Data are presented as Mean ± SEMs. Different superscripts (a, b, c) in each parameter indicate significance (*p* < 0.05).

To investigate the impact of different photoperiods on melatonin, we measured the MT concentrations in the hypothalamus, cecum and breast muscle (Figure 1). As depicted in Figure 1, following treatment with extending light exposure, the concentrations of MT significantly decreased in the hypothalamus, cecum and breast muscle (*p* < 0.05).

### 3.3 Effects of different photoperiods on breast muscle tissue morphology by HE staining

**Figure 2:**
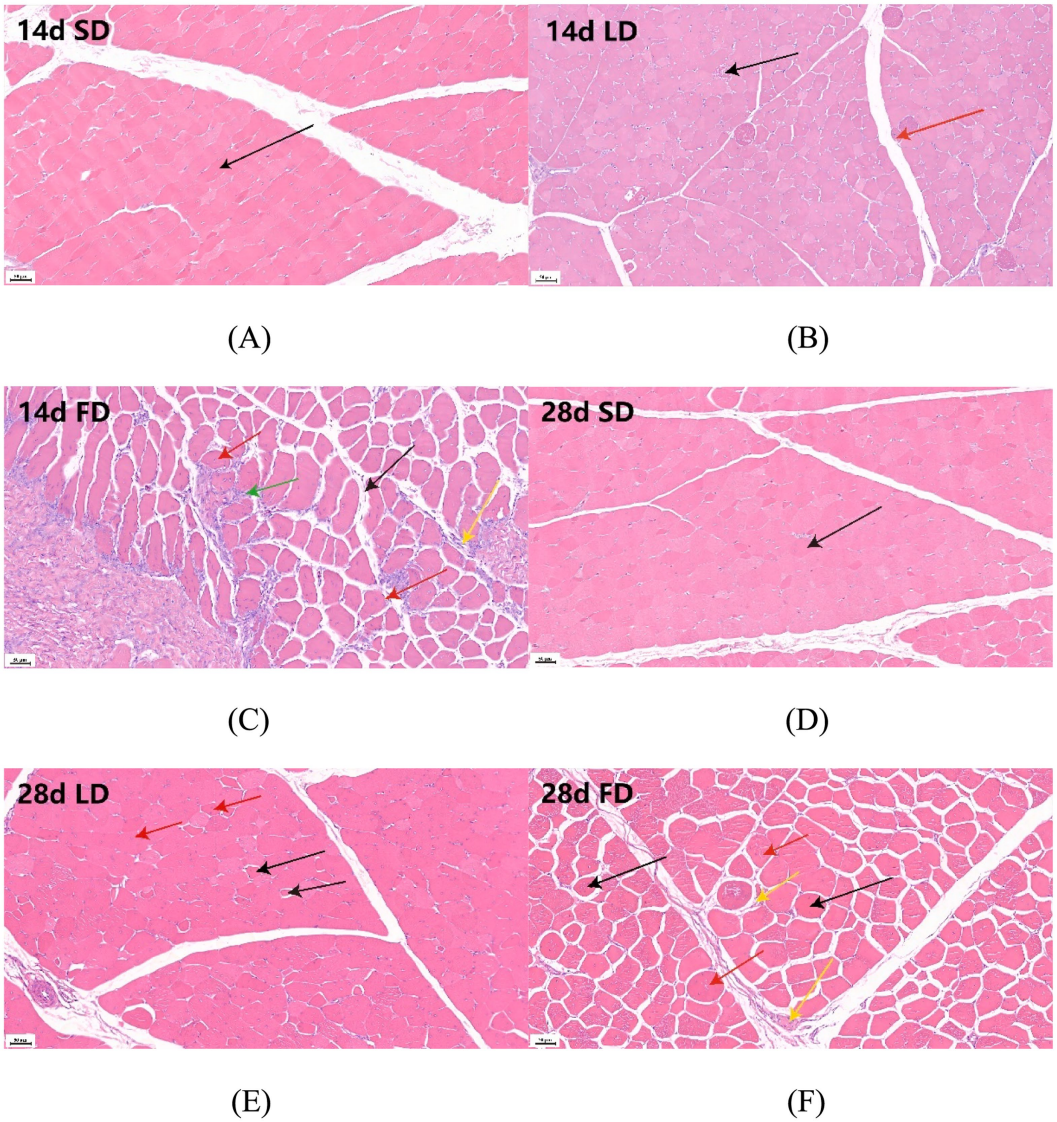
Effects of different photoperiods on the breast muscle morphology of broilers. **(A)** 14d SD group. **(B)** 14d LD group. **(C)** 14d FD group. **(D)** 28d SD group. **(E)** 28d LD group. **(F)** 28d FD group. Figures were 20× magnification. **(A)** The black arrow indicates muscle fiber. **(B)** The black arrow indicates muscular bundle morphology lysis and disappearance; the red arrow indicates intranuclear migration. **(C)** The black arrow indicates an increasing gap; the red arrows indicate intranuclear migration; the yellow arrow indicates fibroblast hyperplasia; the green arrow indicates inflammatory cell infiltration. **(D)** The black arrow indicates muscle fiber. **(E)** The black arrow indicates an increasing gap; the red arrow indicates intranuclear migration. **(F)** The black arrow indicates an increasing gap; the red arrows indicate intranuclear migration; the yellow arrow indicates fibroblast hyperplasia.

As depicted in Figures 2A–C, we observed abnormal organizational structure in both the LD group and the FD group during 14 days. The overall structure of the breast muscle appeared normal in the 14d SD group, with no signs of muscle fiber atrophy or necrosis (black arrow indicates muscle fiber) (Figure 2A). However, the 14d LD group exhibited mild histopathological abnormalities in the breast muscle, including necrosis (black arrow indicates muscular bundle morphology lysis and disappearance) and minor myogenic injury in the muscular bundle (red arrow indicates intranuclear migration) (Figure 2B). Additionally, the 14d FD group showed injuries to the overall structure of the breast muscle, such as extensive muscular bundle injury and atrophy (black arrow indicates increasing gap, red arrows indicate intranuclear migration), muscular bundle interstitium fibrosis (yellow arrow indicates fibroblast hyperplasia), and inflammatory cell infiltration (green arrow) (Figure 2C).

As also shown in Figures 2D–F, the abnormal organizational structure was observed to increase in the LD group and the FD group at 28 days. The overall structure of the breast muscle in the 28d SD group was normal, similar to that of the 14d SD group (Figure 2D). The 28-day LD group exhibited both myogenic injury and atrophy, as indicated by the increasing gap (black arrows) and intranuclear migration (red arrows) (Figure 2E). Similarly, the breast muscle in the 28-day FD group showed abnormalities throughout the day, with myogenic injury and atrophy observed in the muscular bundle, along with increasing gaps (black arrows), intranuclear migration (red arrows), and fibroblast hyperplasia in the muscular bundle interstitium (yellow arrows) (Figure 2F). The above results suggested that extended light exposure could injure the breast muscle morphology with inflammatory cell infiltration.

**Figure 3:**
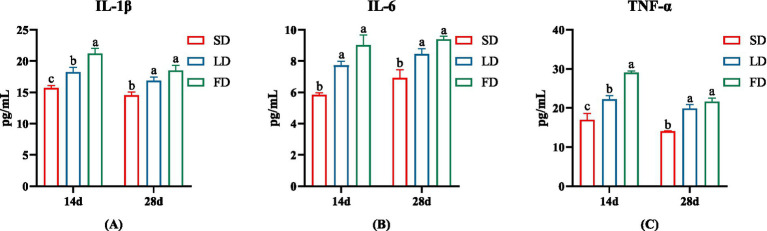
Effects of different photoperiods on inflammatory cytokines concentrations of broilers. **(A)** IL-1β. **(B)** IL-6. **(C)** TNF-*α*. Data are presented as Mean ± SEMs. Different superscripts (a, b, c) in each parameter indicate significance (*p* < 0.05).

To further verify the inflammation levels of the breast muscles, we examined the levels of IL-1 *β*, IL-6 and TNF *α* in the breast muscle. The inflammatory factor concentrations on the breast muscle of broilers significantly increased under extending light exposure. On 14d and 28d, compared with the SD group, the concentrations of IL-1 β, IL-6 and TNF α in breast muscle significantly increased in the LD group and the FD group (*p* < 0.05; Figure 3).

### 3.4 Effects of different photoperiods on cecal microbiota of broilers

**Figure 4:**
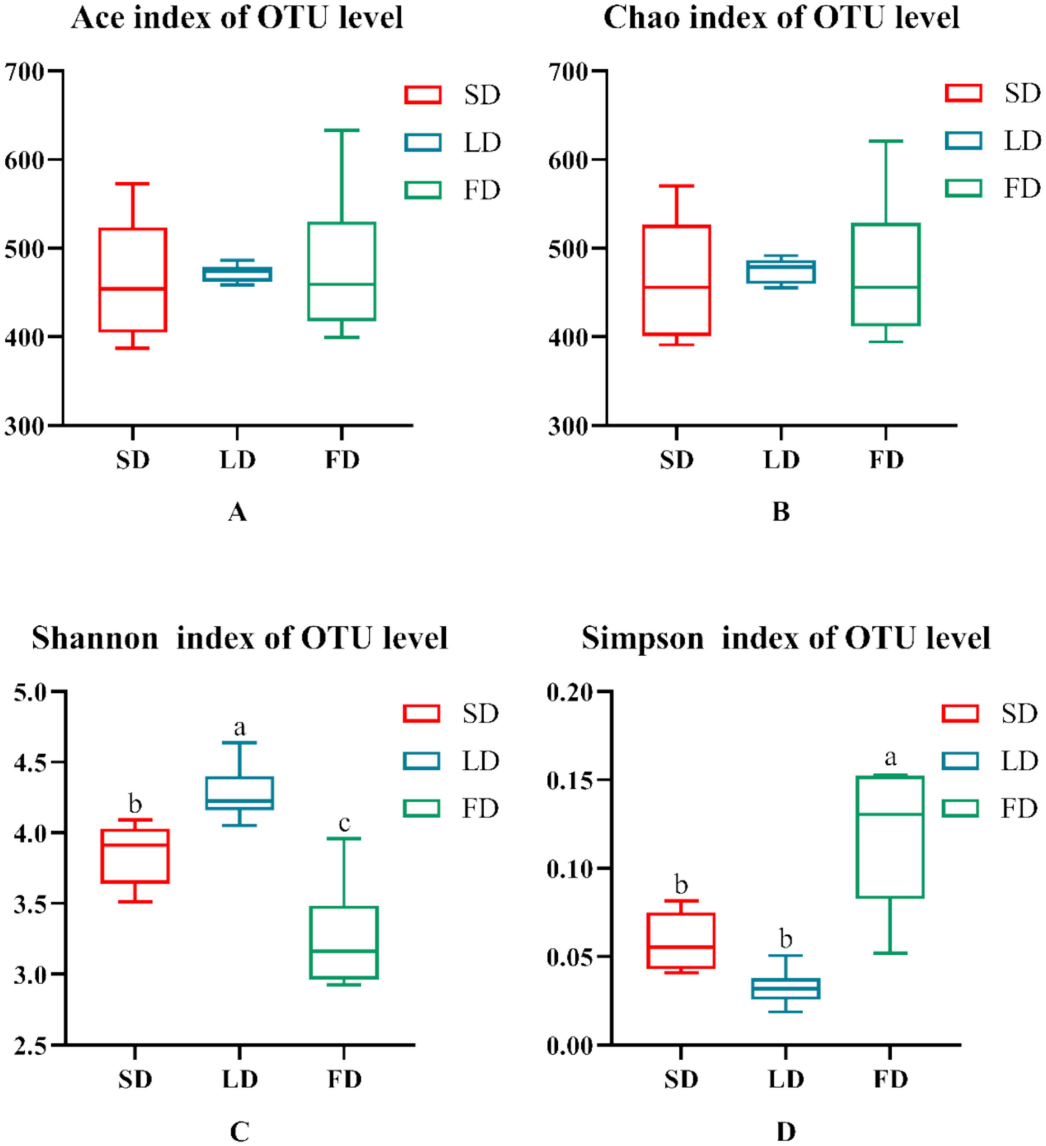
Indexes of alpha diversity of the microbiota. **(A)** Ace. **(B)** Chao. **(C)** Shannon. **(D)** Simpson. Data are presented as Mean ± SEMs. Different superscripts (a, b, c) in each parameter indicate significance (*p* < 0.05).

**Figure 5:**
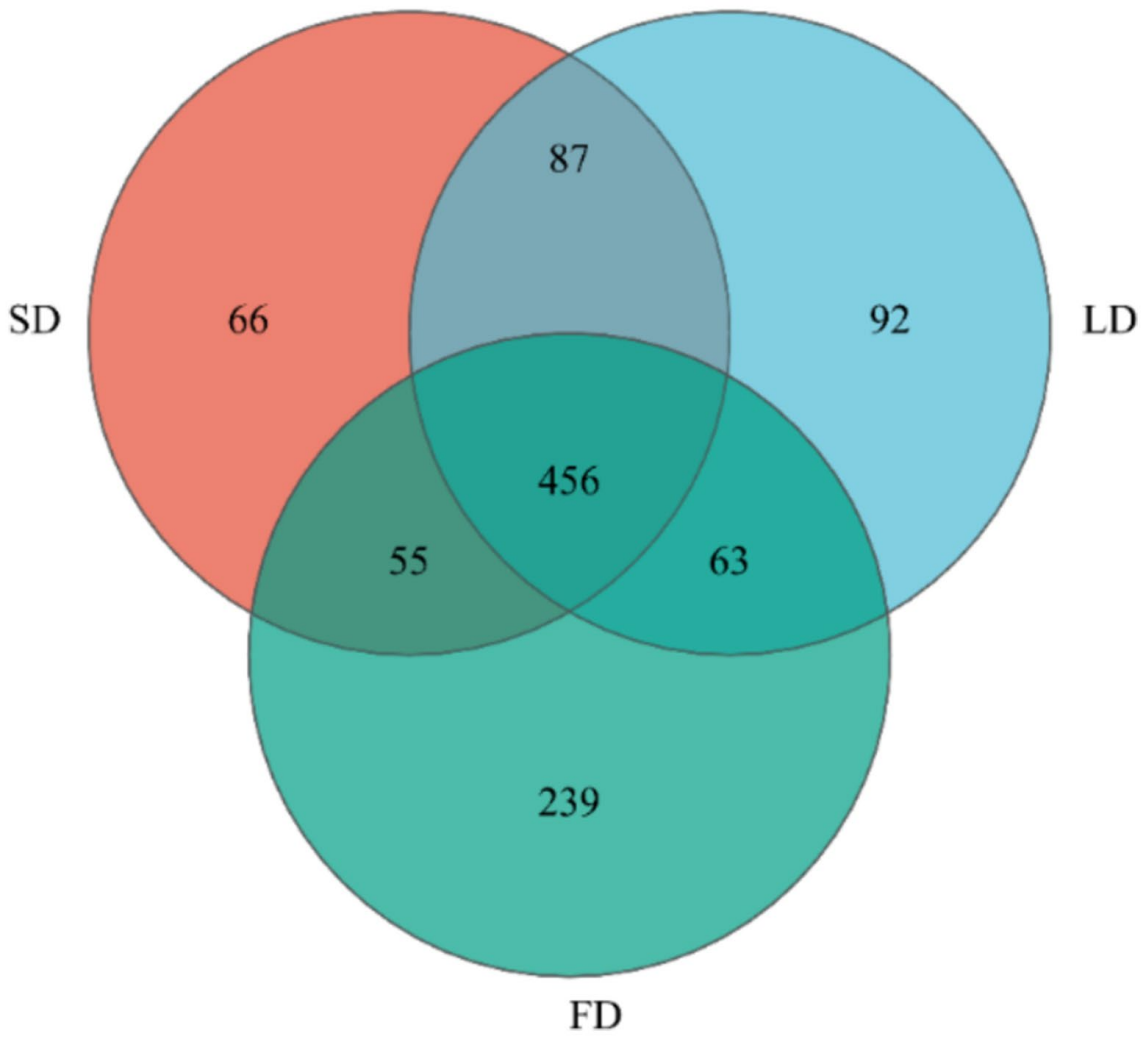
Venn diagram on OTU level.

**Figure 6:**
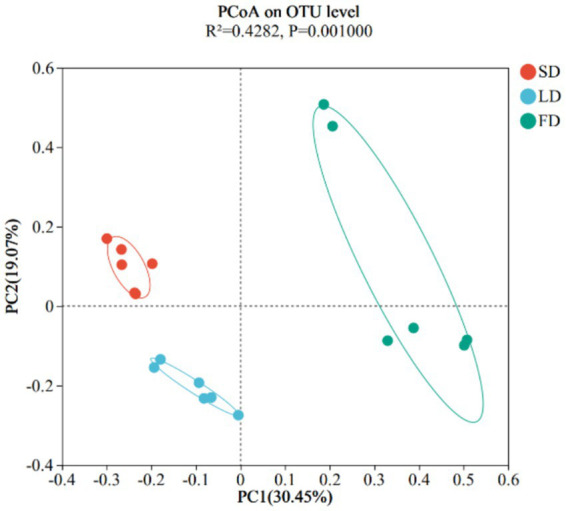
Beta diversity was calculated using Principle Coordinates Analysis plots based on unweighted UniFrac distance.

Finally, we analyzed the composition of cecal microbiota. The ACE index (A), Chao index (B), Shannon index (C), and Simpson index (D) are shown in Figure 4. The results indicated that the indices reflecting species richness (ACE index and Chao index) had no significant effect under different photoperiods (*p* > 0.05). However, the indices of community diversity (Shannon index and Simpson index) were significantly affected (*p* < 0.05) under different photoperiods. These findings suggested that the community diversity exhibited an increase followed by a decrease with the increase of photoperiods. At the OTU level, Venn diagram showed microorganisms found in the cecal content were identified with 664, 698, and 813 OTUs, respectively. (Figure 5). Moreover, the overall structure of the cecal microbiota on the OTU level in the three groups significantly differed (R^2^ = 0.4282, *p* = 0.001), as analyzed by principal coordinate analysis (PCoA) (Figure 6).

**Figure 7:**
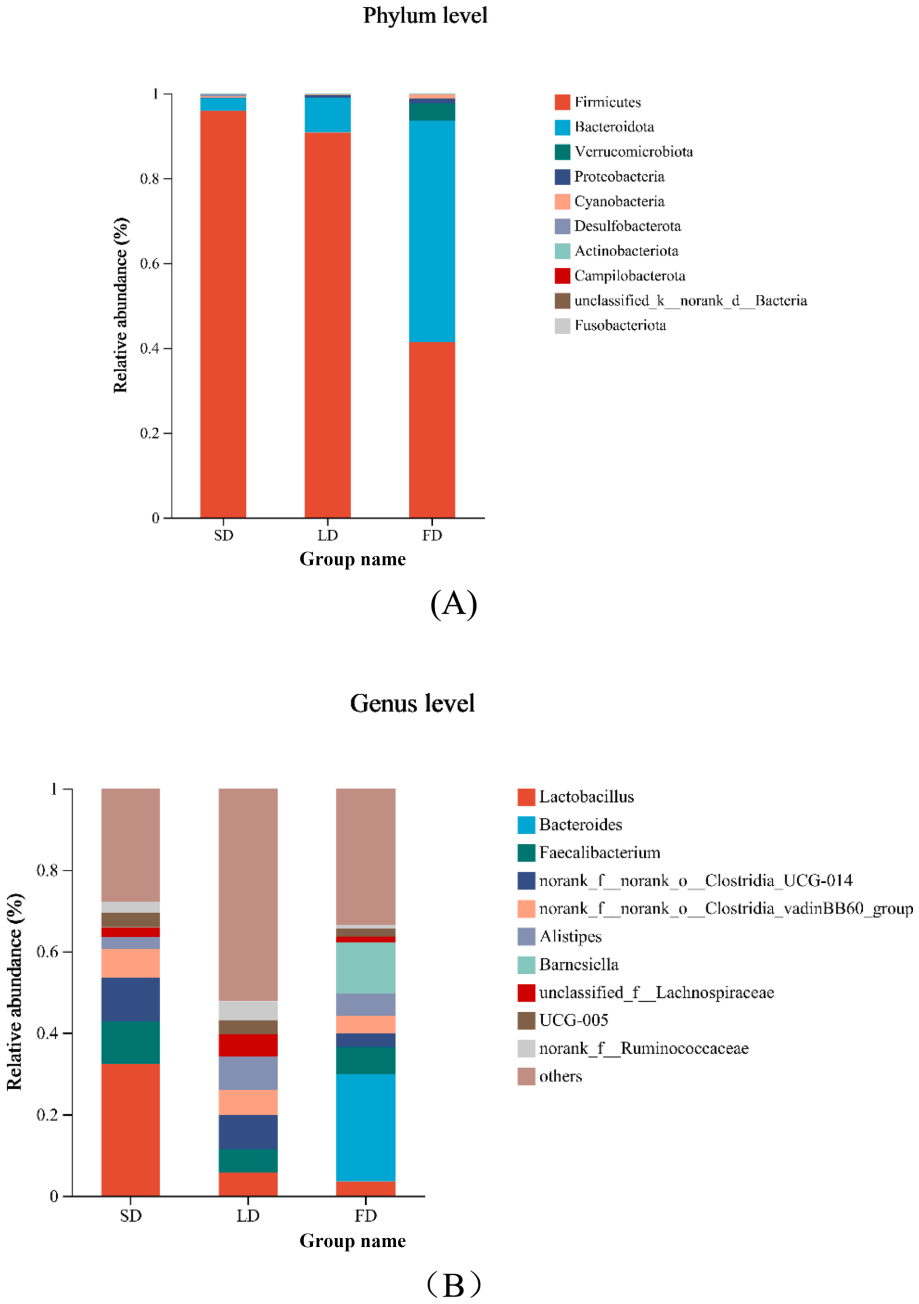
Relative abundance of cecal microbiology composition at the phylum and genus and species in the different photoperiods group. **(A)** Relative abundance of cecal microbiology at the phylum; **(B)** relative abundance of cecal microbiology at the genus.

**Figure 8:**
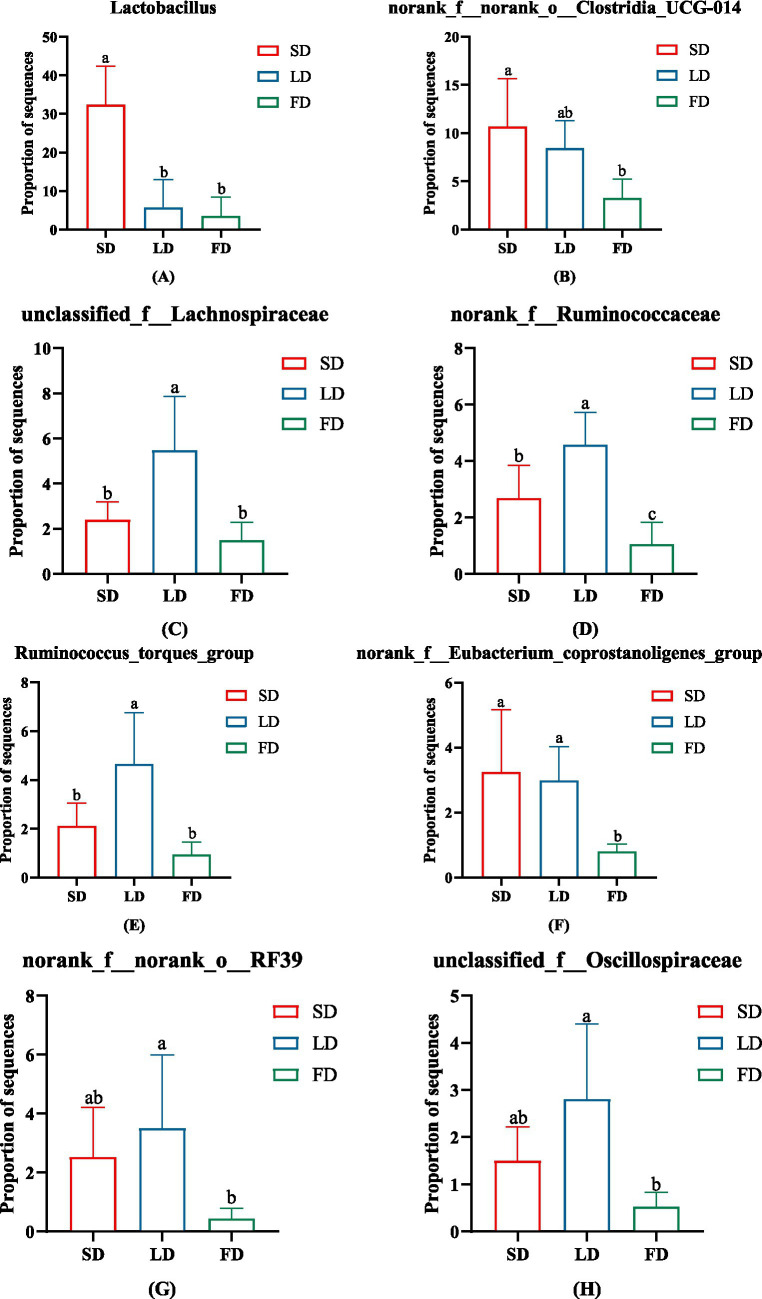
The significant change in the relative abundance of cecal microbiota on genus levels. **(A)** *Lactobacillus*. **(B)** *norank_f__norank_o__Clostridia_UCG-014*. **(C)** *unclassified_f__Lachnospiraceae*. **(D)** *norank_f__Ruminococcaceae*. **(E)** *Ruminococcus_torques_group*. **(F)** *norank_f__Eubacterium_coprostanoligenes_group*. **(G)** *norank_f__norank_o__RF39*. **(H)** *unclassified_f__Oscillospiraceae*. Data are presented as Mean ± SDs. Different superscripts (a, b, c) in each parameter indicate significance (*p* < 0.05).

Taxon-based analysis showed the cecal microbial composition markedly changed on account of different light treatments (Figures 7A,B). Relative abundance analysis of the top 10 phyla in the cecal microbiota was shown in Figure 7A, it was found that *Firmicutes* and *Bacteroidota* were the dominant microbiota, with the relative abundance in the three groups 95.91, 90.75, 41.28, and 2.99%, 8.22, 52.22%, respectively. The relative abundance of *Firmicutes* and *Bacteroidota* was significantly decreased and increased in the FD group, respectively, compared with the SD group and LD group (*p* < 0.05). The FD group significantly increased the relative abundance of *Proteobacteria* in comparison with the SD group (*p* < 0.05). Compared with the LD group, the relative abundance of *Cyanobacteria* had markedly increased in the FD group (*p* < 0.05). Relative abundance analysis of bacteria in the cecal microbiota was shown in Figure 7B, it was found that *Lactobacillus*, *Bacteroides*, *Faecalibacterium*, *norank_f__norank_o__Clostridia_UCG-014*, *norank_f__norank_o__Clostridia_vadinBB60_group*, *Alistipes*, *Barnesiella*, *unclassified_f__Lachnospiraceae*, *UCG-005* and *norank_f__Ruminococcaceae* was the top 10 bacteria with higher relative abundance. To evaluate the impact of different photoperiods on the taxonomic composition of microorganisms in the cecal contents, Kruskal-Wallis H test was employed to determine the genera with significant effects on the three groups. As shown in Figure 8, the results were mainly evaluated for the top 20 bacteria under three different photoperiods. The results indicated that there were 8 bacteria (*Lactobacillus*, *norank_f__norank_o__Clostridia_UCG-014*, *unclassified_f__Lachnospiraceae*, *norank_f__Ruminococcaceae*, *Ruminococcus_torques_group*, *norank_f__Eubacterium_coprostanoligenes_group*, *norank_f__norank_o__RF39*, *unclassified_f__Oscillospiraceae*) was significantly changed under different photoperiods. The relative abundance of *Lactobacillus*, *norank_f__norank_o__Clostridia_UCG-014, norank_f__Eubacterium_coprostanoligenes_group* was markedly reduced with longer photoperiods (*p* < 0.05; Figures 8A,B,F). As the photoperiods increased, the relative abundance of *unclassified_f__Lachnospiraceae*, *norank_f__Ruminococcaceae*, *Ruminococcus_torques_group*, *norank_f__norank_o__RF39* and *unclassified_f__Oscillospiraceae* first enriched and then reduced (*p* < 0.05; Figures 8C–E,G,H).

**Figure 9:**
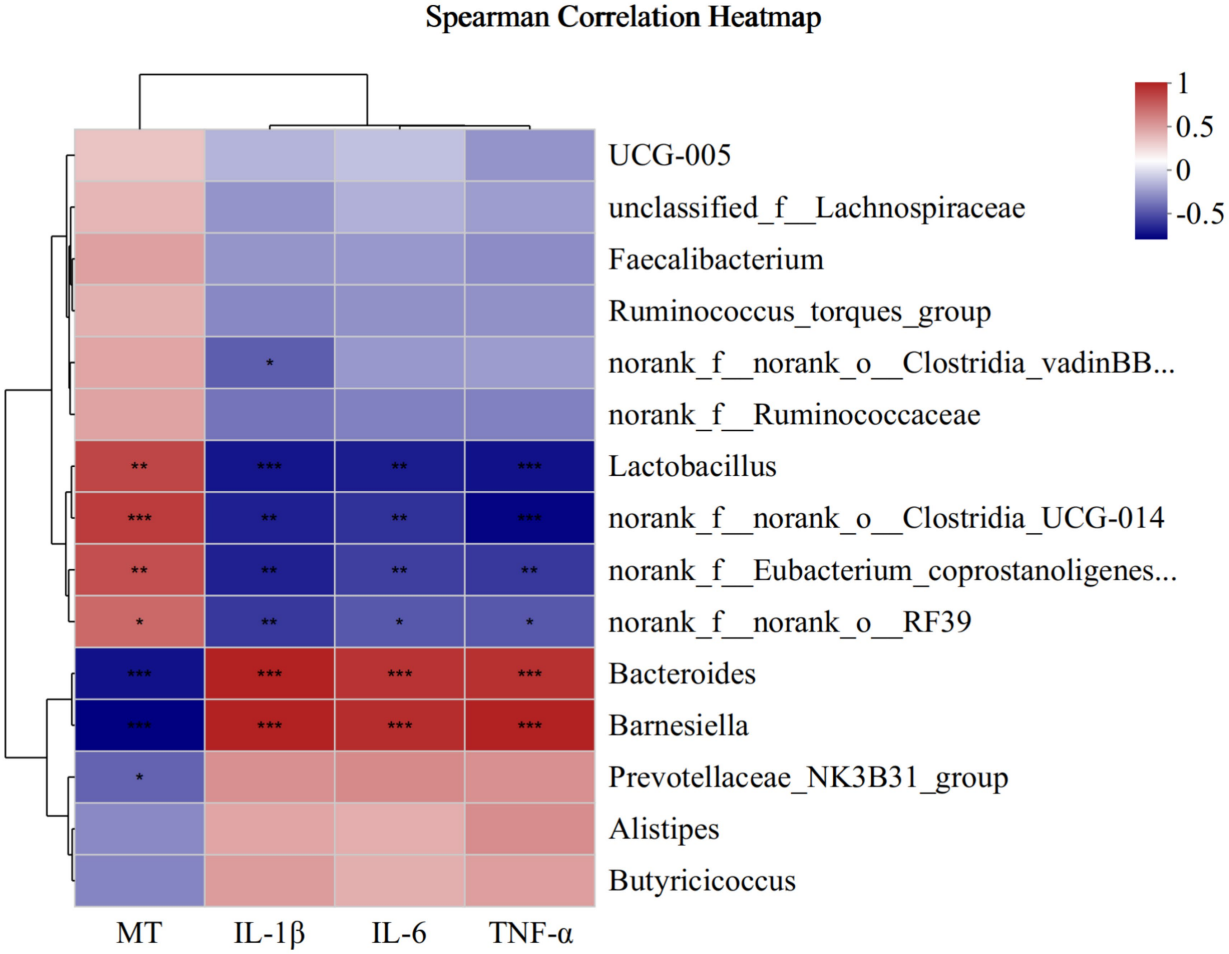
Correlation between microbiota abundance with cecum MT, IL-1β, IL-6 and TNF-α at the genus. * means *p* ≤ 0.05, ** means *p* ≤ 0.01, *** means *p* ≤ 0.001.

To investigate the correlation between microbiota abundance with MT, IL-1β, IL-6 and TNF-*α*, we analyzed that by environmental factor correlation analysis based on Spearman correlation analysis (Figure 9). At the genus level, the relative abundance of *Lactobacillus*, *norank_f__norank_o__Clostridia_UCG-014*, *norank_f__Eubacterium_coprostanoligenes_group* and *norank_f__norank_o__RF39* was a positive correlation with MT and negative correlation with IL-1β, IL-6 and TNF-α, *Bacteroides* and *Barnesiella* was negative correlation with MT and positive correlation with IL-1β, IL-6 and TNF-α. Furthermore, *Prevotellaceae_NK3B31_group* had a negative correlation with MT, and norank_f__norank_o__Clostridia_vadinBB60_group had a negative correlation with IL-1β.

## 4 Discussion

The main aim of this study was to explore the association between MT and cecal microbiota under extended light exposure as well as the potential regulatory pathways mediating the effects of different photoperiods on the breast muscle morphology of broilers. Recent studies have shown that continuous long photoperiods may negatively affect broiler health and welfare (Tuell et al., 2020). The results indicated that exposure to long photoperiods increased growth rate but decreased FE, impaired breast muscle morphology and significantly increased the concentrations of IL-1β, IL-6 and TNFα in breast muscle. Furthermore, MT was found to be related to cecal microbiota under different photoperiods, which also affected breast muscle morphology and inflammation of broilers.

Previous research has shown that long photoperiods enhance the growth rate and overall chicken production performance (Zhang et al., 2022; Ingram et al., 2000). Similarly, this study found LD and FD photoperiods increased the growth rate of broilers. During the 4-week trial period, the FE was significantly decreased following FD treatment. This finding was consistent with results from previous studies, which uncovered faster growth rates and higher FCR (Olanrewaju et al., 2006; Shynkaruk et al., 2022).

Notably, broilers are highly sensitive to changes in light, with studies showing that light stimulation enhances the inhibition of MT secretion from the pineal gland via the superior cervical ganglion by the suprachiasmatic nucleus of the hypothalamus under light stimulation, whereas in the absence of light stimulation at night, the pineal gland secretes MT (Shichida and Matsuyama, 2009). Nighttime light exposure decreases MT secretion (Yang et al., 2024), and in this study, we found that MT concentrations in the hypothalamus, cecum and breast muscle of broilers decreased significantly under extended light exposure. This is consistent with previous studies (Yang et al., 2024), demonstrating that extended light exposure may decrease MT levels.

Modern broilers are often genetically selected for higher breast muscle yield and faster growth rates. However, they are particularly vulnerable to oxidative stress (Xing et al., 2022; Pontes et al., 2023). Guob et al. reported that a longer photoperiod may increase oxidative stress levels (Guo et al., 2010). In this study, the concentrations of IL-1β, IL-6 and TNFα increased in the breast muscle accompanied by inflammatory cell infiltration in the breast muscle of broilers under FD treatment. Moreover, the injury to the breast muscle was positively correlated with the IL-1β, IL-6 and TNFα levels. Therefore, we speculated that the injury was induced by an inflammatory response following long photoperiod which might be related to oxidative stress. In summary, the results of this study showed that prolonged exposure to light exposure caused morphological injury and inflammation in the breast muscles of broiler chickens, especially under the FD treatment.

Cecal microbiota are important modulators of various pathological processes. The cecum harbors a wide range of symbiotic bacteria, which participate in microbial fermentation and prevent pathogen colonization (Huang et al., 2018; Guarner and Malagelada, 2003). Therefore, the diversity and composition of cecal microflora mature and stabilize as the broilers age, reaching a stable state in 21 days (Zhou et al., 2021). In this study, we characterized the cecal microbiota composition of 33-day-old broilers following exposure to different photoperiods. The alpha richness index (diversity within a community) of cecal microbiota first increased and then reduced with longer photoperiods. This was consistent with findings from previous studies exploring the effects of 12.5 L:11.5D and 16 L: 8D photoperiods, suggesting that the obtained observations were induced by the effects of long photoperiods on broiler metabolism (Wang et al., 2018). At the phylum level, Firmicutes was the dominant bacterial phylum across the three photoperiods. The dominant bacterial phylum in the FD group changed to *Bacteroidota* instead of *Firmicutes*, and *Bacteroidota*, *Proteobacteria*, and *Cyanobacteria* were significantly elevated in the FD group. Researchers have shown that *Firmicutes* are beneficial bacteria, and a decrease in their relative abundance increases the risk of systemic inflammation and development of certain diseases (Samaddar et al., 2022; Qu et al., 2022). Numerous pathogens belonging to the *Proteobacteria*, may cause diseases to broilers (He et al., 2022). Therefore, the observed difference in the FD group may be explained by the increased effect of the treatment on cecal microbiota, which enhanced cecal microbial disorders and increased the relative abundance of harmful bacteria. At the genus level, *Lactobacillus* was enriched in the SD group. The genus *Lactobacillus* contains probiotics which has been reported to be decreased under long photoperiods (Song et al., 2020; Zhang et al., 2022). A study found that the relative abundance of *Lactobacillus* influenced the development of inflammation in broilers (Yang et al., 2020; Khalique et al., 2019), suggesting that exposure to long photoperiods may suppress the abundance of probiotics and trigger cecal microbiota disorder, possibly by increasing inflammation levels. Interestingly, we found that the relative abundance of *Ruminococcus_torques_group* initially increased and then reduced with the prolongation of light exposure. The *Ruminococcus_torques_group* has been associated with inflammation and the occurrence of various health issues, including neurological diseases and abdominal fat deposition (Zheng et al., 2020; Blacher et al., 2019; Chen et al., 2022; Xiang et al., 2024; Lyu et al., 2021). However, the function of *Ruminococcus_torques_group* on broilers and its changes under different photoperiods has not been clarified. The decrease in *Ruminococcus_torques_group* in the FD treatment may be attributed to the severe disruption of cecal microbial composition. Notably, the *Ruminococcus_torques_group* belongs to *Firmicutes*, which was reduced by 46.63% in the FD treatment, accompanied by a decrease in the relative abundance of *Ruminococcus_torques_group*. The ratio of *Ruminococcus_torques_group* to *Firmicutes* increased, suggesting enhanced relative abundance. Altogether, our results indicated that prolonged exposure to light significantly altered the cecal microbial composition in broiler chickens by reducing beneficial bacteria and enriching the relative abundance of harmful bacteria, causing the cecal microbiota disorder.

The brain-gut axis involves the bidirectional communication between the nervous, endocrine and immune systems (Liu et al., 2018). Numerous studies have shown that MT regulates gut microbiota composition (Iesanu et al., 2022; Ma et al., 2020). Studies have reported that alterations in the intestinal microbial composition could affect inflammation (Niu et al., 2022). To further explore the correlations among MT, cecal microbiota and inflammation in breast muscle under different photoperiods, we analyzed the association of cecal microbiota with changes in MT, IL-1β, IL-6 and TNF-*α* levels. It was observed that the composition of cecal microbiota was significantly influenced by changes in MT, IL-1β, IL-6 and TNF-α concentrations. *Lactobacillus*, which is the most important probiotic in the cecum of broilers, was positively correlated with MT levels and negatively correlated with IL-1β, IL-6 and TNF-α levels. On the other hand, *Bacteroides* was found to enhance inflammation in mice (Li et al., 2020), and negatively correlated with MT levels and positively with IL-1β, IL-6 and TNF-α levels in this study. In comparison, *Barnesiella* has been reported to play dual roles in enteric infections: exerting colonization resistance against pathogenic invaders, while also inducing detrimental effects on the host (Bornet and Westermann, 2022). In this study, the relative abundance of Barnesiella increased as the concentrations of inflammatory markers increased and the MT levels decreased. This suggested that the decreased MT levels resulted in the down-regulation of the beneficial bacteria and an increased in harmful bacteria, inducing inflammation in the breast muscle of broilers.

Notably, the brain-gut axis is increasingly being recognized and studied. Here, we found that prolonged exposure of mice to light weakened the MT levels thereby reducing the number of beneficial bacteria and enriching the number of harmful bacteria, which caused morphological injury and inflammation in the breast muscle of broilers. Therefore, we speculated that the MT-gut axis may mediate the morphological injury and inflammation induced by prolonged exposure to light in the breast muscle of broilers. Furthermore, *Lactobacillus* was significantly reduced under extended light exposure, and closely related to MT, IL-1β, IL-6 and TNF-α levels, indicating that this bacterial genus may have an important regulator function on MT and inflammation; however, this requires further research.

In summary, this study demonstrated that prolonged exposure to light enhanced the growth rate of broilers. However, it reduced the FE, induced morphological injury and inflammation in the breast muscle of broilers, suppressed the MT levels and disrupted the cecal microbial structure. Moreover, the regulatory pathways mediating the effects of extended light exposure included inflammatory signaling in breast muscle driven by the MT-gut axis. These findings have important implications for future research into the role of MT and cecal microbiota in breast muscle morphology injury and inflammation under different photoperiods. To improve the meat quality of broilers, we recommend that the 18 L:6D photoperiod may be optimal compared with the 12 L:12D and 24 L:0D photoperiod.

## Data Availability

The data presented in the study are deposited in NCBI/SRA repository, accession number PRJNA1190345.

## Footnotes

^1^ https://cloud.majorbio.com
